# Serum Amyloid P Therapeutically Attenuates Murine Bleomycin-Induced Pulmonary Fibrosis via Its Effects on Macrophages

**DOI:** 10.1371/journal.pone.0009683

**Published:** 2010-03-12

**Authors:** Lynne A. Murray, Rogerio Rosada, Ana Paula Moreira, Amrita Joshi, Michael S. Kramer, David P. Hesson, Rochelle L. Argentieri, Susan Mathai, Mridu Gulati, Erica L. Herzog, Cory M. Hogaboam

**Affiliations:** 1 Promedior, Inc., Malvern, Pennsylvania, United States of America; 2 Department of Pathology, University of Michigan Medical School, Ann Arbor, Michigan, United States of America; 3 Internal Medicine, Pulmonary and Critical Care Division, Yale University School of Medicine, New Haven, Connecticut, United States of America; University of Pittsburgh, United States of America

## Abstract

Macrophages promote tissue remodeling but few mechanisms exist to modulate their activity during tissue fibrosis. Serum amyloid P (SAP), a member of the pentraxin family of proteins, signals through Fcγ receptors which are known to affect macrophage activation. We determined that IPF/UIP patients have increased protein levels of several alternatively activated pro-fibrotic (M2) macrophage-associated proteins in the lung and monocytes from these patients show skewing towards an M2 macrophage phenotype. SAP therapeutically inhibits established bleomycin-induced pulmonary fibrosis, when administered systemically or locally to the lungs. The reduction in aberrant collagen deposition was associated with a reduction in M2 macrophages in the lung and increased IP10/CXCL10. These data highlight the role of macrophages in fibrotic lung disease, and demonstrate a therapeutic action of SAP on macrophages which may extend to many fibrotic indications caused by over-exuberant pro-fibrotic macrophage responses.

## Introduction

The substantial morbidity and mortality associated with pulmonary fibrosis makes this heterogeneous group of diseases an important area of research. This group of disorders encompasses the interstitial lung diseases (ILD), idiopathic pulmonary fibrosis (IPF/UIP), radiation-induced pulmonary fibrosis, scleroderma-induced lung disease (SSc-ILD), and drug-induced lung toxicity. Collectively these pathologies are associated with uncontrolled matrix deposition, collagen production, apoptosis, and alveolar destruction. Airway based diseases can also develop fibrosis and lead to chronic airway obstruction and persistent gas exchange abnormalities. Despite its clinical importance, an underlying common mechanism contributing to fibrosis remains obscure.

A monocyte-derived cell type associated with the maintenance and progression of ILD, notably IPF/UIP, is the alternatively activated (M2) macrophage. This phenotype of macrophage is the predominant macrophage found in the lungs of IPF/UIP patients [1]. These cells express IL13Rα2 and signaling though this high affinity IL13 receptor results in TGFβ_1_ expression, thus promoting the fibrotic milieu [2]. Further, M2 macrophages also express higher levels of scavenging receptors CD163, mannose receptor (MRC-1, CD206) and macrophage scavenging receptor (MSR-1) as they differentiate from monocytes [3], [4].

M2 macrophages are capable of synthesizing pro-fibrotic mediators, but this cell type is inefficient at supporting the host defense provided by the classical M1 macrophage [3]. This alteration in macrophage phenotype in IPF/UIP patients may explain why these patients succumb to repeated bouts of pulmonary infections or exacerbation of disease, which has been correlated with a more rapid decline in lung function and ultimately death [5]. Therefore, regulating the phenotype of this cell is an attractive therapeutic strategy for lung fibrosis, as this cell type produces high levels of pro-fibrotic cytokines and growth factors *in vitro*, which may contribute towards the continuation of the fibrotic milieu.

Serum amyloid P (SAP), a member of the pentraxin family of proteins has been shown to inhibit fibrosis in a number of organ sites during preclinical animal models, which in part is due to an inhibition of the differentiation of peripheral blood mononuclear cells into CD45^+^/collagen I^+^ cells called fibrocytes [6], [7], [8]. SAP binds to Fcγ receptors [9] and the anti-fibrotic activities of SAP have been shown to be mediated through Fcγ receptors [10] which affect peripheral blood monocyte differentiation and activation states.

We initially phenotyped monocytes from IPF/UIP patients and detected increased CD163 expression, which correlated with increased M2 associated proteins in the circulation and lung. We next hypothesized that the anti-fibrotic therapeutic benefit mediated by SAP during bleomycin-induced lung fibrosis is via directing monocyte/M2 macrophage activation.

## Results

### Enhanced alternative macrophage phenotype in IPF/UIP patient lung and circulation

To determine the potential for altered monocyte/macrophage phenotype in IPF/UIP, we initially assessed the levels of various mediators in the circulation of patients with stable and progressive IPF/UIP. 25 subjects were recruited. Of these, thirteen had IPF/UIP and twelve were control subjects. There was no difference in the control and IPF/UIP subjects in terms of sex, race, age, and comorbid conditions including obesity, coronary disease, or renal disease. While no control subjects smoked, seven IPF/UIP subjects had a significant smoking history (p<0.0005, Table 1). IPF/UIP patients were then stratified into “Progressive” or “Stable” based on their clinical outcome at one year. Seven patients qualified as “progressive” and six qualified as “stable.” This included: Significant (10%) decline in FVC in the 12 months following enrollment, new oxygen requirement, hospitalization or treatment for exacerbation of IPF/UIP, and death by any cause. Using these criteria, seven IPF/UIP patients qualified as “progressive” and six were “stable.” There was no difference in the baseline characteristics of groups including race, sex, age, comorbid conditions, biopsy proven diagnosis, and lung function. These data are shown in Table 2.

**Table 1:** IPF vs control.

Category		IPF n = 13	Control n = 12	Composite P Value per Category
Sex	Female	2 (15.4)	4 (33.3%)	0.38
	Male	11 (84.63%)	8 (66.7%)	
Smoking History	Ever	5 (38.5%)	0	0.002
	Never	8 (61.5%)	0	
Race	Nonwhite	4 (30.7%)	2 (8.3%)	0.64
	White	9 (61.3%)	10 (91.7%)	
Obese	Nonobese	10 (76.9%)	10 (83.3%)	1.0
	Obese	3 (23.1%)	2 (16.7%)	
Age	Years	66.5±10.9	59.25±16.0	0.19
Coronary disease	Incidence	1 (7.6%)	1 (8.3%)	1.0
Kidney disease	Incidence	0	0	NS

Continuous variables are expressed as mean ± standard deviation.

**Table 2:** Progressive vs Stable IPF.

Comparison		Progressive (n = 7)	Stable (n = 6)	Composite P Value per Category
Sex	Female	1 (14.3%)	1 (16.7%)	1.0
	Male	6 (85.7%)	5 (83.3%)	
Smoking History	Ever	3 (42.9%)	2 (33.3%)	1.0
	Never	4 (57.1%)	4 (66.7%)	
Race	Nonwhite	3 (42.9%)	1 (16.7%)	0.56
	White	4 (57.1%)	5 (83.3%)	
Obese	Nonobese	5 (71.4%)	5 (83.3%)	1.0
	Obese	2 (28.6%)	1 (16.7%)	
Age	Years	61.7±11.7	72.0±7.30	0.09
Coronary Disease	Incidence	1 (14.3%)	0 (100%)	1.0
Kidney disease	Incidence	0 (100%)	0 (100%)	NS
IPF Diagnosis Basis	Biopsy	5 (71.4%)	5 (83.3%)	1.0
	FVC (% predicted)	54.7±10.3	67.5±13.5	0.08
	DLCO (% predicted)	52.8±19.4	58.7±21.3	0.63

Continuous variables are expressed as mean ± standard deviation.

**Figure 1:**
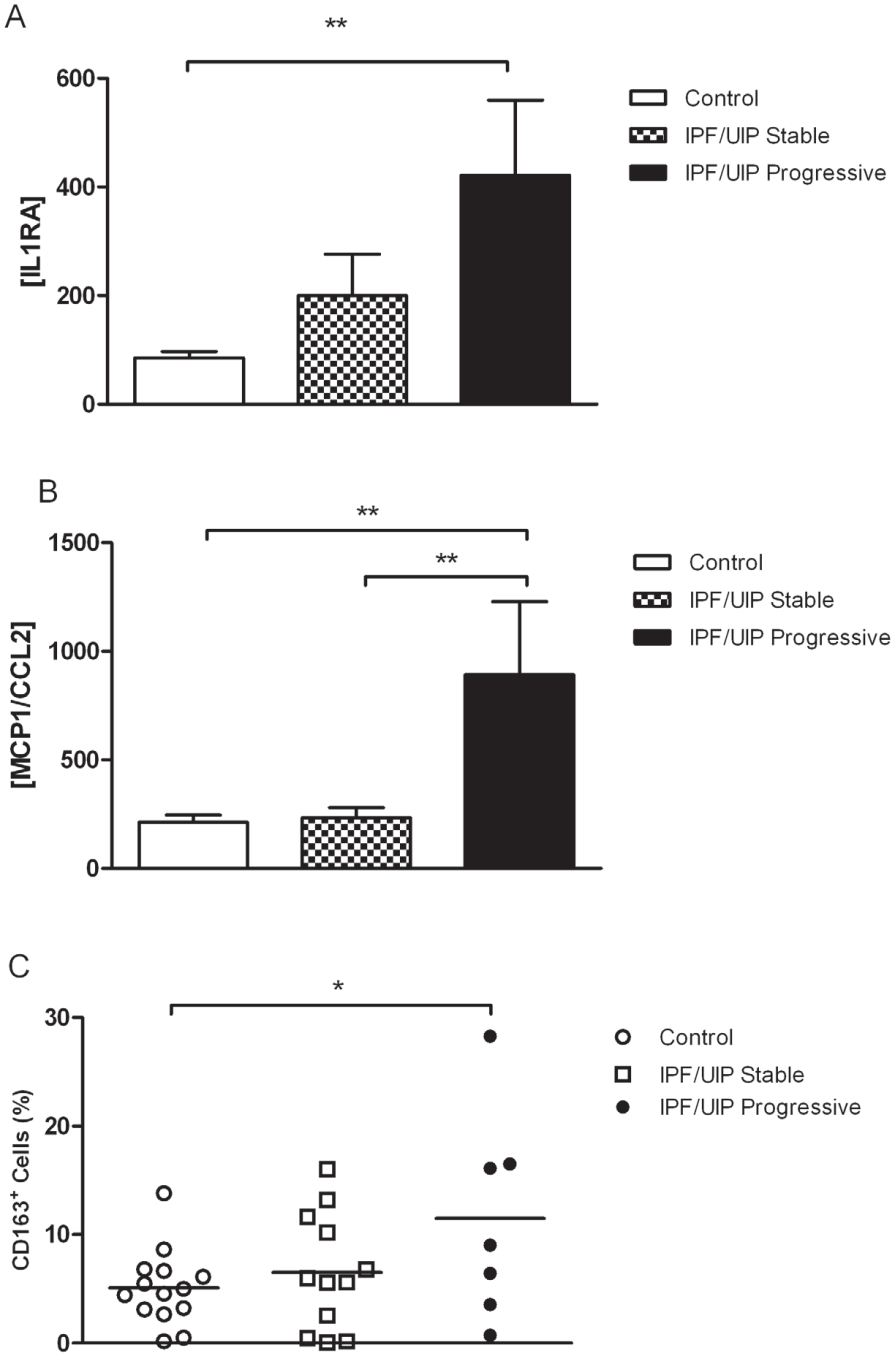
Increased M2 macrophage phenotype and markers in the circulation of IPF/UIP patients. (A–B) Plasma levels of IL1RA (A) and MCP1/CCL2 (B) for controls (*n* = 12), stable IPF/UIP (*n* = 6) and progressive IPF/UIP (*n* = 7) as described in Tables 1and 2 were determined using Luminex™ technology. (C) CD163 expression on CD14^+^ monocytes isolated from IPF/UIP patients (filled bar, *n* = 12) and healthy age-matched controls (open bar, *n* = 8) was assessed by flow cytometry. Mean ± s.e.m. *P≤0.01 **P≤0.005 ***P≤0.001 significance using Mann Whitney analysis with Bonferroni correction.

We analyzed plasma from IPF/UIP and control subjects and focused on the two monocyte-associated markers IL1RA and MCP1/CCL2 (Fig. 1). Here we determined that patients with IPF/UIP had significantly increased plasma IL1RA (Fig. 1 A) and MCP1/CCL2 (Fig. 1 B) in comparison to healthy age-matched control patients. When stratifies into stable versus progressive, IPF/UIP patients with stable disease only trended towards an increase in IL1RA compared to controls, however there was no increase in MCP1/CCL2 in the circulation of IPF/UIP patients with stable disease. Interestingly, IPF/UIP patients with progressive disease had significantly greater levels of MCP1/CCL2 compared to stable disease patients (Fig. 1 B). Flow cytometric analysis indicated that a greater percentage of isolated CD14+ monocytes also expressed CD163 in IPF/UIP patients in comparison to healthy age-matched controls (Fig. 1 C). In addition, CD163+ monocytes were increased in patients with progressive versus stable disease.

**Figure 2:**
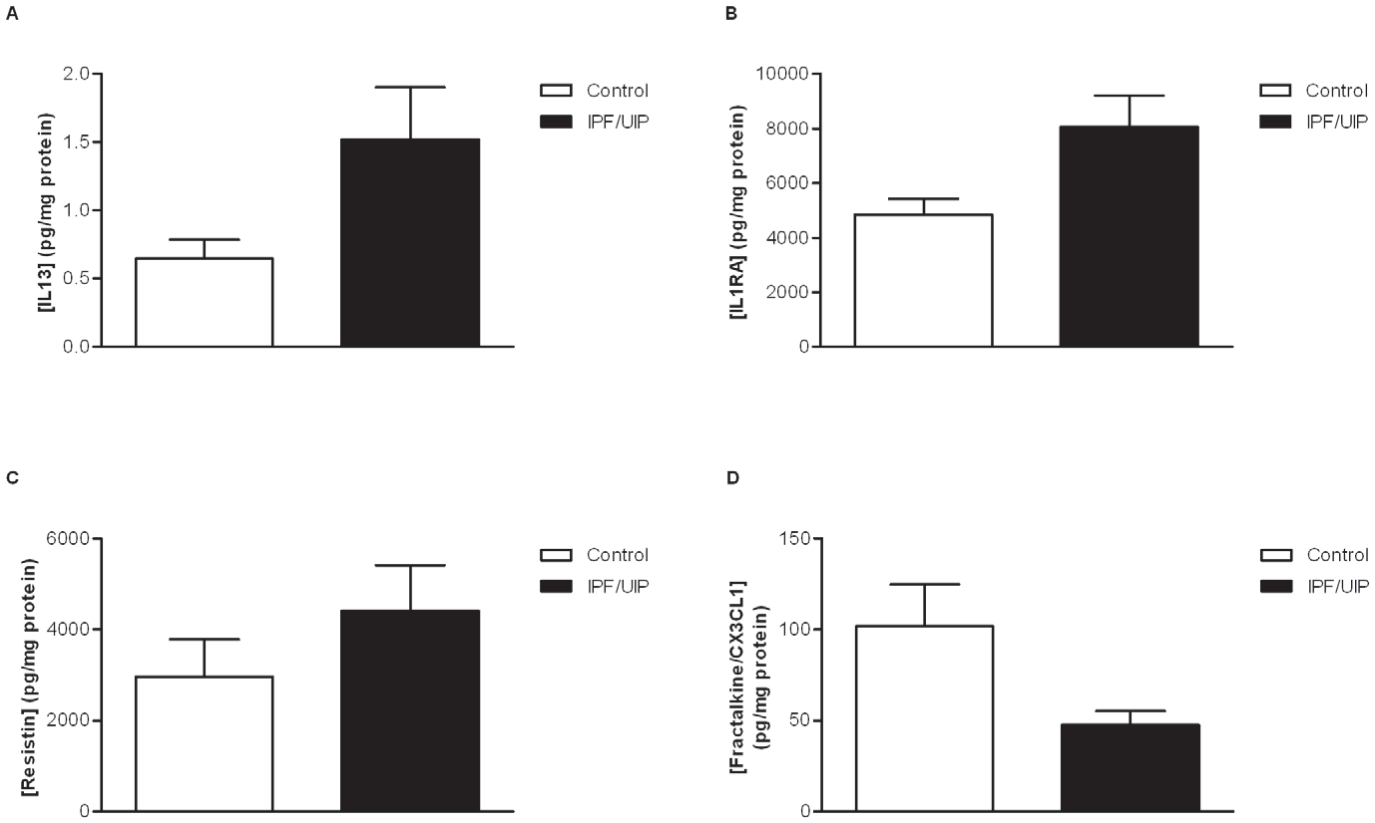
Enhanced M2 monocyte/macrophage phenotype in IPF/UIP. (A–D) Levels of macrophage-associated mediators measured in lung tissue biopsies taken from IPF/UIP patients (filled bars) or from the normal margins of lung tumors following resection (open bars). M2 macrophage-associated IL13 (A), IL1RA (B) and resistin (C); and M1-associated fractalkine/CX3CL1 (D) from non-fibrotic (*n* = 11) or IPF/UIP (*n* = 13) lung tissue were assessed using Luminex technology. *P≤0.05 significance using Mann Whitney analysis.

Previous reports have demonstrated that M2 macrophages are present in the lungs of IPF/UIP patients [1], [11]. Using Luminex™ technology we measured the levels of specific macrophage-associated mediators at the protein level in lysates of whole lung tissue obtained from biopsy samples from IPF/UIP patients taken at time of diagnosis and compared it to control lung tissue from the normal margins of lung tumor resections. For this study we measured markers associated with M1/M2 macrophages, namely IP10/CXCL10, MIP1α/CCL3 and fractalkine/CX3CL1 for M1 and IL4, IL13, IL1RA and resistin for M2 macrophage phenotype. We observed significantly elevated levels of IL13 and IL1RA in IPF/UIP lung tissue (Fig. 2 A,B). We also detected a trend towards an increase in resistin levels in the lung of IPF/UIP patients compared to non-IPF/UIP, although the levels were not significantly different (Fig. 2 C). In contrast, the lungs of IPF/UIP patients had significantly decreased levels of the M1 macrophage-associated protein fractalkine/CX3CL1 (Fig. 2 D). There was no difference in IP10/CXCL10, MIP1α/CCL3 or IL4 levels in IPF compared to non-fibrotic tissue (data not shown).

### Attenuated alternative macrophage activation mediated by SAP in established bleomycin-induced lung fibrosis

**Figure 3:**
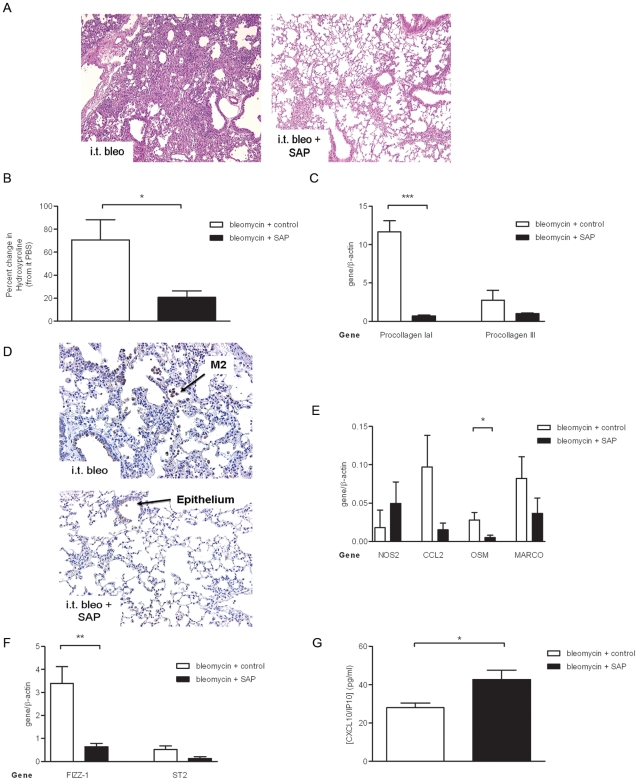
Attenuated bleomycin-induced lung fibrosis and M2 macrophage phenotype with SAP treatment. Mice challenged with intratracheal bleomycin (0.05 U) on day 0 and dosed with SAP (6 mg/kg, i.p. filled bars) or control (PBS, i.p., open bars) on days 11–20 were evaluated at day 21 for gross morphological changes visualized in H&E stained lung sections (A); collagen generation quantitated by hydroxyproline analysis (B); procollagen IaI and procollagen III gene expression quantitated and normalized to β-actin by branched DNA technology (C). Macrophage phenotype in the lung assessed following SAP treatment by immunolocalizing IL13Rα2 (D), gene transcript levels determined by branched DNA technology at day 21 for NOS2, CCL2, oncostatin (OSM) and MARCO (E), and FIZZ1 and ST2 (F). (G) Plasma IP10/CXCL10 levels determined by Luminex technology. Bars represent the mean ± s.e.m. of a minimum of *n* = 4 per group. Original magnification, ×20. *P≤0.05 significance using Student's t-test.

Our previous data show that macrophages in the lung are largely of an M2 phenotype by day 11 after bleomycin [12]. A previous study in has demonstrated that murine serum-derived SAP reduces fibrosis in a mouse intratracheal bleomycin model [7]. Here, as expected, human SAP also significantly reduced the histopathological extent of bleomycin-induced collagen deposition (Fig. 3 A) and the generation of collagen at the protein (Fig. 3 B) and gene level (Fig. 3 C).

To extend the initial published findings, we next evaluated the phenotype of macrophages in bleomycin model, we initially stained lung sections for IL13Rα2 expression. In the lung, IL13Rα2 is highly expressed on both the epithelium and on M2 macrophages [13]. We stained lung sections with anti-IL13Rα2 and found comparable levels of epithelial IL13Rα2 expression between vehicle and SAP-treated bleomycin challenged mice at day 21 (Fig. 3 D). However, there was a reduction in the number of IL13Rα2+ alveolar macrophages in the lungs of SAP treated mice in comparison to vehicle control treated mice (Fig. 3 D). Analysis of a wider panel of M2-associated genes using branched DNA technology in the lungs of bleomycin challenged mice treated with SAP indicated that SAP also reduced the expression of the M2-associated proteins MARCO (Fig. 3 E), ST2 and FIZZ-1 (Fig. 3 F); as well as attenuating the bleomycin-induced pro-fibrotic mediators CCL2 (MCP1/JE) [14], and oncostatin M (OSM) [15] (Fig. 3 E).

We also found a trend towards an increase in M1 macrophage-associated NOS2 expression following SAP treatment (Fig. 3 E). Moreover, SAP treatment increased the level of the M1 macrophage-associated chemokine IP10/CXCL10, in the circulation of mice treated with SAP, in comparison to control treated bleomycin-challenged animals (Fig. 3 G).

**Figure 4:**
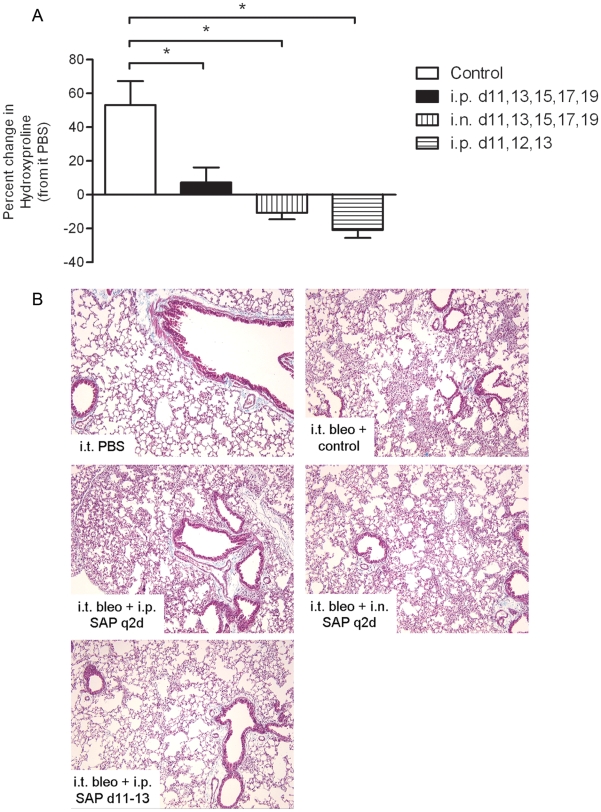
Local and abbreviated SAP treatment significant reduces bleomycin-induced collagen generation and deposition. Mice were challenged with intratracheal bleomycin (0.03 U) on day 0 and dosed with SAP (6 mg/kg) every other day on days 11–19 intraperitoneally (i.p., filled bar) or intranasally (i.n., down-hatched bar); or dosed with SAP (10 mg/kg, i.p.) on days 11,12,13 only (cross-hatched bar); or dosed with control (P5S, i.n., open bar) every other day on days 11–19. Collagen was quantitated biochemically (A) and visualized histologically (B). Original magnification, ×20. Bars represent the mean ± s.e.m. of a minimum of *n* = 5 per group. *P≤0.05 significance using Mann Whitney t-test.

Due to the profound effect on alveolar macrophages, we next compared the ability of locally delivered SAP to reduce bleomycin-induced collagen deposition with intranasal SAP dosing, to systemically delivered SAP. Here systemically or intranasally dosed recombinant human SAP (rhSAP) significantly reduced bleomycin-induced collagen generation and deposition when the protein was dosed every other day from day 11 (Fig. 4 A,B). Interestingly, dosing mice systemically on days 11, 12 and 13 had the most pronounced effect on attenuating bleomycin-induced increases in collagen deposition which suggests that effects on circulating or extrapulmonary monocytes may be at least partially responsible for the ameliorative effects seen in these models.

## Discussion

Alternatively activated macrophages have been described in the lungs of IPF patients [1], however the correlation with disease severity and their presence in the periphery has not. Here we determined an elevated M2 background in these patients, both in the lung and circulation. We then tested the hypothesis that the pro-fibrotic activation of monocytes/macrophages seen in many types of human pulmonary fibrosis is central to the progression of disease pathology. A previous study has highlighted the potential for the short pentraxin SAP to inhibit bleomycin induced lung fibrosis, in part through an inhibition of monocyte to fibrocyte differentiation *in vitro* [6] and accumulation *in vivo* [7]. We extended these studies to assess a more global effect on lung monocyte biology. Our results demonstrate that the modulation of macrophage activation in established bleomycin-induced lung fibrosis inhibited bleomycin-induced increases in collagen deposition.

To determine the clinical significance of specific macrophage subtypes during lung fibrosis we initially assessed the environment of the fibrotic lung. During IPF/UIP, there is an M2 skewed milieu, as indicated by elevated IL13, IL1RA and resistin; and decreased M1 macrophage-associated fractalkine/CX3CL1 in comparison to the non-fibrotic normal margins of lung tumour resection tissue. The likely source of IL13 in the IPF/UIP lung is the lymphocyte. However, we have attempted to stain for IL13 in IPF lung sections and found this to be technically challenging. Moreover, the role of T cells during lung fibrosis is controversial [16] and further studies with potential advances in technology to help delineate the cellular source of IL13 would add to our current understanding.

Similar results with IL1RA were seen in the peripheral blood, where we found increased IL1RA in IPF/UIP. As the non-fibrotic tissue used in this study is the normal margins of lung tumor resections, this tissue may have contained tumour-associated macrophages which have been reported to be skewed to a M2 phenotype [17]. Therefore, our results indicate that the macrophage phenotype in the IPF/UIP lung is even more dramatically skewed towards this alternatively activated phenotype.

We next detected elevated M2 macrophages in the circulation of IPF/UIP patients and this was further increased in patients that have progressive lung disease. Interestingly, we also found elevated M2 associated proteins in the circulation, suggesting an overall elevated M2 background in these patients. Monocytes are capable of differentiating into a multitude of cell types including fibrocytes and alternatively activated M2 macrophages [18]. Due to the pronounced extent of remodeling in the lungs of IPF patients prior to any therapeutic intervention, we assessed the ability of SAP to attenuate established bleomycin-induced lung fibrosis buy initiating dosing at day 11. In this model at this time point, significant collagen deposition has occurred [19]. The anti-fibrotic effect mediated by SAP on fibrocytes has been demonstrated however, its role in pulmonary macrophage activation has been unknown. We hypothesized that similar effects would be seen on another subset of pro-fibrotic cells derived from circulating monocytes, namely the M2 macrophage. Indeed, our data indicate broad effects on monocyte cell fate that extend beyond fibrocyte differentiation and increase the repertoire of diseases for which this protein may be therapeutically significant.

Recent evidence has suggested that alveolar macrophages can derive from both the circulating monocyte pool as well as an intermediate lung macrophage [20]. We observed comparable efficacy when SAP was dosed systemically or locally to the lung. Interestingly, the mice that were only dosed with SAP on days 11, 12 and 13 had the most profound amelioration of fibrosis at day 21. Pharmacokinetic analysis of human SAP in mice indicated that the circulating half-life after intraperitoneal administration for this protein was approximately 8 hours (data not shown). This suggests that the durability of response exceeds the circulating properties of the protein and may be via a modulation of cell phenotype. Further, studies using labeled SAP indicated that intranasally delivered SAP persisted with the lung at least 96 hours after dosing, whereas systemically delivered SAP localized to the liver (data not shown). Taken together these data suggests that the duration of beneficial effects mediated by SAP extends beyond its systemic half-life, and that the site of action may be in the circulation as well as in the lung. This therefore further demonstrates the potential utility of SAP to treat human disease.

Analysis of the macrophage phenotype during *in vivo* models indicated that SAP inhibits the M2 phenotype in the lungs of mice with pulmonary fibrosis. Extensive marker analysis of whole lung tissue indicated an attenuation of multiple M2 macrophage-associated markers, including IL13Rα2, and the scavenging receptor MARCO. SAP significantly reduced other key pro-fibrotic mediators produced by M2 macrophages including, FIZZ-1 (found in inflammatory zone 1), CCL2 [14], OSM [15] and ST2 [21]. We also detected enhanced resistin levels in the lungs of IPF/UIP patients. The murine equivalent of resistin is FIZZ-1 (found in inflammatory zone 1) and has been previously associated with fibrotic regions of lung fibrosis models [22]. Taken together, M2 associated markers elevated in IPF/UIP, namely IL13, CCL2 and resistin, were all reduced *in vivo* with SAP treatment.

SAP also promoted an increase in the M1-associated marker, NOS2, as well as increasing the M1-associated chemokine CXCL10/IP10. The CXCL10/IP10 finding is of particular interest as this chemokine, as well as being associated with an M1 macrophage phenotype, also has other anti-fibrotic activities including inhibiting fibroblast recruitment [23], recruiting IFNγ-producing NK cells [24] and reducing aberrant angiogenesis [25]. Overall this suggests an SAP-mediated inhibition of the M2 macrophage phenotype and that this may extend to the clinical setting.

In addition to specific effects on fibrosis *per se*, modulation of macrophage phenotypes could potentially decrease the rate and severity of pulmonary infections and fatal exacerbations, which are thought to occur in IPF/UIP patients due to an impairment in innate immunity. Lastly, these findings could extend to other diseases characterized by the M2 phenotype such as asthma and COPD.

## Materials and Methods

### Patients

All studies were performed with Human Investigations Committee approval at Yale University School of Medicine or University of Michigan School of Medicine. For the plasma and monocyte studies, inclusion criteria were: All patients were greater than 18 years of age who had been diagnosed with IPF/UIP based on the ATS/ERS 2002 guidelines were eligible for enrollment. Exclusion criteria included current or recent use (within 2 months) of immunosuppression or experimental therapy; chronic infection such as HIV, tuberculosis, or hepatitis; known pulmonary hypertension, COPD, or asthma; unstable cardiovascular, renal or neurologic disease; and inability to provide informed consent. Healthy, age-matched controls who self-identified as normal were recruited from the greater New Haven area. Following enrollment and written informed consent, demographic data concerning age, race, sex, and co-morbid conditions were collected on all subjects. In addition, data regarding restrictive ventilatory defect (decreased forced vital capacity, or FVC) and diffusion impairment (decreased diffusion capacity for carbon monoxide, DLCO) and how the diagnosis of IPF/UIP (biopsy or CT scan) was made were obtained from chart abstraction on the patients with IPF/UIP. After one year of follow up patient charts were reviewed for the following IPF/UIP-relevant outcomes: Significant (10%) decline in FVC in 6 months following enrollment, new oxygen requirement, hospitalization or treatment for exacerbation of IPF/UIP, and death by any cause. Patients who met one or more of these criteria were considered “progressors” and those who did not were considered “Stable” (Table 1).

### Bleomycin-induced pulmonary fibrosis model

All *in vivo* bleomycin studies were conducted according to University of Michigan's IACUC regulations and protocols. To induce pulmonary fibrosis, female C57Bl/6 mice were treated with high dose (0.05 U/mouse) or standard dose (0.03 U) of bleomycin (Blenoxane, Sigma, St. Louis, MO) intratracheally on day 0, as previously described [25], [26].

### Human serum amyloid P

Human serum amyloid P (SAP) was purchased from EMD biosciences as human serum derived SAP frozen in PBS without sodium azide preservative. Recombinant human serum amyloid P was produced by Promedior as PRM-151 from CHO-S cell culture. PRM-151 was provided as a PBS preservative free solution. SAP was dosed either intraperitoneally (i.p.) or intranasally (i.n.) as stated in the Figure Legend.

### Protein and mRNA analyses

For protein analysis, lung biopsy tissue from IPF/UIP patients or from the normal margins of lung tumor resections, or lungs from mice were homogenized in anti-protease buffer (Roche Diagnostics Corp., Indianapolis, Indiana, USA) and processed as previously described [26]. Protein levels were measured by bead based Luminex™ analysis or specific ELISA. For gene analysis, total RNA was obtained using TRIzol reagent (Invitrogen) according to the manufacturer's instructions. In some instances, mediator levels were normalized to protein content which was determined using a standard Bradford protein assay. Gene expression levels were quantitated using branched-DNA-technology-based QuantiGene Reagent System (Panomics), according to the manufacturer's protocols. Transcript levels of fibrosis and macrophage-related genes (procollagen I, procollagen III, NOS2, CCL2, oncostatin M, MARCO, FIZZ1 and ST2) were normalized to β-actin mRNA.

### Collagen assessment

Collagen levels were determined in lung homogenates using an established hydroxyproline assay as previously described [12].

### Histologic analysis

Formalin-fixed and paraffin-embedded lung sections were stained with hematoxylin and eosin to assess gross morphology or Mallory's trichrome stains to visualize collagen deposition.

### Immunohistochemical localization of IL13Rα2 in histological tissue sections

Formalin-fixed and paraffin-embedded lung sections were analyzed for immunohistochemical localization of IL13Rα2 as previously described [12]. Sections were blocked with normal rabbit serum (Vectorstain ABC-AP kit, Vector Laboratories, Burlingame, CA). Rabbit anti-mouse IL13Rα2 antibody (R&D Systems) and control normal rabbit IgG were diluted in PBS to a final concentration of 5 µg/ml. A secondary rabbit anti-goat biotinylated antibody (Vector Laboratories) was added to each section, then each slide was thoroughly washed and receptor localization was revealed using HRP-Dab Staining Kit (R&D Systems).

### *In vitro* monocyte isolation and flow cytometry

Thirty mLs of peripheral blood was drawn and processed as previously described [11]. Briefly, following density gradient separation with Ficoll-paque (Stem Cell Technologies) cells were stained for flow cytometric analysis of CD14 (Miltenyi) and CD163 (BD Pharmingen) expression. Flow cytometry and cell sorting was performed using a BD FACSCalibur. Data were analyzed using Flow Jo v 7.5 software (TreeStar, Inc).

### Statistics

Normally distributed data are expressed as means ± s.e.m. and assessed for significance by Student's *t* test or ANOVA as appropriate. Data that were not normally distributed were assessed for significance using the Wilcoxon rank sum test of Mann Whitney U test where appropriate. Patient demographics were compared using Student's t-test or Mann Whitney analysis as appropriate. Categorical variables were compared using Fisher's exact test. Multi-analyte comparisons were performed using Mann-Whitney analysis. P values were determined for multiple comparisons using the Bonferroni correction.
